# From childhood seizures to a neuroscientific legacy: marking the bicentennial of Dom Pedro II

**DOI:** 10.47626/1516-4446-2025-4325

**Published:** 2025-12-18

**Authors:** Marleide da Mota Gomes, Antonio E. Nardi

**Affiliations:** 1 Programa de Pós-Graduação de Psiquiatria e Saúde Mental, Instituto de Psiquiatria, Faculdade de Medicina, Universidade Federal do Rio de Janeiro (UFRJ), Rio de Janeiro, RJ, Brazil; 2 Laboratório de História da Psiquiatria, Neurologia e Saúde Mental, Instituto de Psiquiatria, UFRJ, Rio de Janeiro, RJ, Brazil; 3 Academia Brasileira de Ciências, Rio de Janeiro, RJ, Brazil; 4 Academia Nacional de Medicina, Rio de Janeiro, RJ, Brazil

As the world commemorates the bicentennial of Emperor Pedro II of Brazil (1825-2025), his visionary promotion of scientific progress calls for fresh appreciation. Known equally as the “Orphan of the Nation” and “the Magnanimous,” his often-overlooked contributions include pioneering support for the development of neuroscience in Brazil^1^-^7^ – an intellectual legacy that grows more significant with time.

This letter seeks to highlight a striking and often overlooked paradox: how an emperor’s own neurological challenges and relentless scientific curiosity contributed decisively to the shaping of Brazil’s neuroscientific landscape.^1^,^2^ From an early age, Pedro II was immersed in a rigorous education encompassing literature, languages, the arts, and natural sciences, a foundation that would steer him toward meaningful dialogues with some of the foremost scientific minds and institutions of his time. His membership in international academies and enduring correspondence with eminent researchers offer a rare window into the global circulation of knowledge in the 19th century and its intersections with the Brazilian imperial court. Dom Pedro II’s intellectualism transcended national borders, making him a pioneer of cultural diplomacy in 19th-century Latin America. His engagement with American and European thinkers – particularly through literature and science – challenged the insularity of Brazilian elites and laid groundwork for future transnational exchanges.^8^ His legacy endures as that of a scholar-king who valued knowledge over power.

The Emperor’s early life was marked by profound personal losses, particularly his mother’s death in 1826 and his father’s abdication and departure in 1831, leaving the young prince to navigate an isolated childhood against a backdrop of political turbulence. Crowned at just 15, Pedro II would go on to reign until 1889, distinguishing himself as an unusually science-minded monarch committed to intellectual freedom, scientific advancement, and humanitarian causes. These formative adversities appear to have ignited an insatiable intellectual appetite that encompassed literature, history, technology, and the natural sciences.^1^-^6^

From his youth, Dom Pedro II cultivated enduring connections with Brazil’s leading medical institutions – particularly the Faculty of Medicine of Rio de Janeiro and the Imperial Academy of Medicine (precursors to today’s UFRJ Medical School and National Academy of Medicine). These relationships proved vital during his childhood struggles with convulsive seizures, exemplifying the 19th century’s intricate ties between imperial power and medical science. Among the notable physicians who shaped this relationship, two figures stand out: Cláudio Velho da Motta Maia, the emperor’s lifelong personal physician, and Joaquim Cândido Soares de Meirelles, a transformative leader in Brazilian medicine.^9^,^10^

Motta Maia, later honored as Count of the Empire, became indispensable to Pedro II’s wellbeing. As both a distinguished professor at the Faculty of Medicine and active reformer of medical education, his dual role as court physician and scientific advisor reflected medicine’s growing prestige in imperial Brazil. His loyalty extended beyond professional duty – he voluntarily accompanied the deposed emperor into exile after 1889, though this devotion ironically led contemporaries to accuse him of undue influence over the monarch’s decisions.

Equally significant was Joaquim Cândido Soares de Meirelles, who presided over the Imperial Academy of Medicine during its crucial transformation from medical society to formal academy (1829-1848). His leadership during the institution’s 1835 reorganization, witnessed by the young Pedro II, and subsequent appointment as Physician to the Imperial Chamber cemented medicine’s institutional role in imperial governance. Together, these physicians embodied the dynamic intersection of medical progress and state power that characterized Pedro II’s reign.

A defining episode in Pedro II’s scientific life occurred in 1876, when he visited Emil du Bois-Reymond’s pioneering electrophysiology laboratory in Berlin.^3^ There, he observed experiments in muscle electrical stimulation and recorded his impressions in travel diaries, displaying particular fascination with Amazonian species such as the *Electrophorus electricus* (electric eel). His keen observations hinted at the promise of Brazil’s rich biodiversity to inform universal neurological principles. Notably, in 1881, Du Bois-Reymond wrote to Pedro II requesting live specimens of electric fish for comparative studies in bioelectricity.

Apparently motivated by this interaction, Pedro II established Brazil’s first physiology laboratory in Rio de Janeiro in 1880. Far from being a ceremonial gesture, the initiative strategically positioned the country within contemporary neurophysiological debates. Under the leadership of French-trained physiologist Louis Couty and Brazilian physician João Baptista de Lacerda, the laboratory became a center for what might today be termed decolonial neuroscience.^4^ Among its significant contributions was groundbreaking research on curare, a neuromuscular blocking agent traditionally used by Indigenous Amazonian peoples. In collaboration with Alfred Vulpian’s laboratory in Paris, these studies laid the foundation for modern pharmacological applications of muscle relaxants in surgical practice. Remarkably, Pedro II insisted that Indigenous knowledge be explicitly acknowledged in these findings – a notably progressive stance within the colonial scientific traditions of his time.

His ethical convictions extended beyond laboratory research. In 1884, Pedro II declined Louis Pasteur’s request to test experimental rabies vaccines on Brazilian prisoners.^5^ This principled refusal represents one of the earliest recorded instances of formal research ethics in the Global South, reflecting the Emperor’s belief that scientific progress must be inseparable from moral accountability. Yet, this ethical vigilance did not curtail medical innovation. In 1888, he financed the creation of Rio de Janeiro’s Pasteur Institute – the first such establishment outside France –, demonstrating his conviction that scientific rigor and humanistic values could, and should, be mutually reinforcing.

Pedro II’s personal relationship with neurological illness was equally consequential.^1^,^2^ He experienced sporadic seizures during childhood, most likely attributable to a benign epilepsy syndrome, particularly given a family history of febrile and afebrile seizures. In adulthood, he was diagnosed with diabetic neuropathy by Jean-Martin Charcot during a consultation in Paris, a diagnosis that embedded him within the rapidly evolving neurological discourse of the era. Retrospective assessments suggest that his late-life symptoms – including fatigue, mood disturbances, and cognitive decline – would today be consistent with vascular cognitive impairment, encompassing cerebrovascular disease and neurodegenerative processes.

His sustained collaboration with neurologist Charles-Édouard Brown-Séquard is particularly illustrative of how personal medical concerns spurred meaningful scientific partnerships.^6^ The Emperor met with Brown-Séquard on multiple occasions during his European tours and they maintained an active correspondence throughout his reign and subsequent exile. Together, they explored galvanic therapies for neuralgia in Empress Teresa Cristina and monitored Pedro II’s own neurological condition with galvanometers.

By the twilight of his reign, Pedro II had evolved from a royal patron of the sciences to a committed participant in clinical and experimental neurology – an early forerunner of what we might now term translational medicine. His bicentennial provides a valuable occasion to reassess the contemporary relevance of his life and work. In an era ever more attentive to the ethical, social, and cultural dimensions of neuroscience, Pedro II’s example reminds us that enduring scientific innovation is often born not from detachment, but from empathy, civic engagement, and a commitment to the common good.

Figure 1 visually chronicles the remarkable scientific and humanitarian legacy of Pedro II, whose 49-year reign transformed Brazil through unprecedented advancements in education, infrastructure, and medical science. Ascending to the throne at just 15 years old, the emperor championed progress through tangible innovations – expanding telegraph and railway networks while establishing pioneering institutions like the astronomical observatory and research laboratories at São Cristóvão Palace. His most enduring medical contribution came at age 16 when he decreed the creation of Latin America’s first psychiatric hospital, the Hospício Pedro II (1842-1852).^7^ This neoclassical landmark on Rio’s Praia da Saudade revolutionized mental healthcare with its humane treatment principles, its significance still embodied today by the emperor’s statue standing before what is now the Federal University of Rio de Janeiro’s Forum. Beyond national borders, Pedro II’s intellectual pursuits found expression in his 1888 meeting with neurology pioneer Jean-Martin Charcot at Aix-les-Bains – captured in a reimagined photograph with his personal physician Cláudio Velho da Motta Maia.^2^ This transnational dialogue continued until the emperor’s death in Paris, where Charcot and Charles Bouchard joined Motta Maia in signing the death certificate now archived at Brazil’s National Academy of Medicine. Even in memorialization, Pedro II’s unique stature endured: as both a member of the Paris Academy of Sciences and correspondent with global institutions, his 1891 funeral attracted international attention, memorialized in *Le Petit Journal’s* contemporary illustration as a monarch whose legacy bridged continents through science and reform.

Through the institutions he founded, his intellectual partnerships, and the enduring symbols of his rule, Pedro II’s vision continues to resonate – a testament to the enduring power of humanist ideals in shaping nations and minds alike. At his bicentennial, Pedro II emerges not solely as a sovereign, but as a pioneering figure in Brazil’s neuroscientific history, whose enduring commitment to ethically grounded, globally engaged, and culturally aware science remains instructive for the fields of contemporary neurosciences.

## Figures and Tables

**Figure 1:**
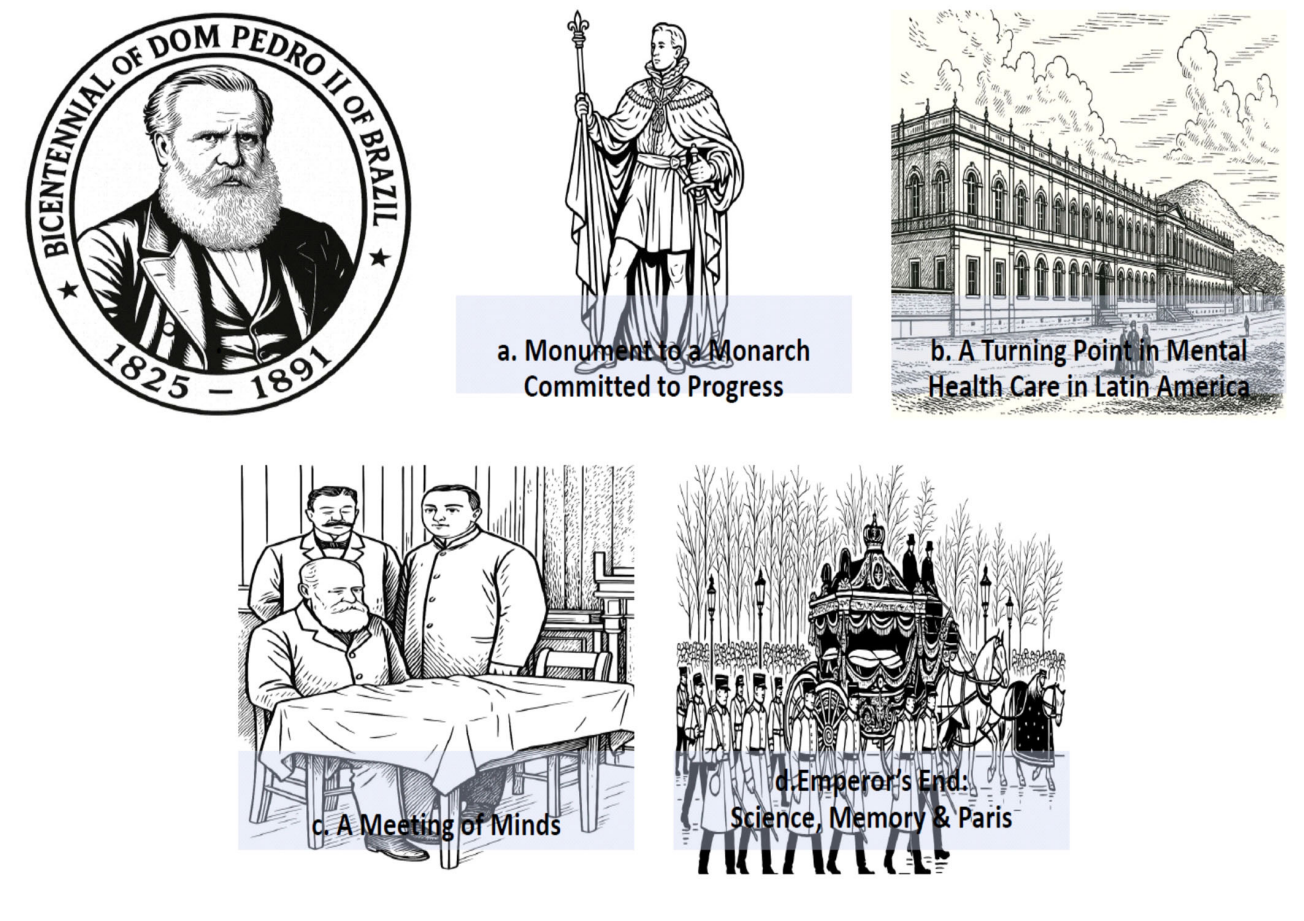
Commemorating Pedro II’s Bicentennial (1825-2025), this composite image pays tribute to Emperor Pedro II of Brazil’s enduring legacy in healthcare innovation and scientific diplomacy. The illustrations highlight his seminal contributions to mental health care, intellectual collaborations, and cultural influence, culminating in a solemn homage at his Parisian funeral. The images have been artistically reimagined in a minimalist style by the first author (MMG) using AI tools, based on sources from Wikipedia (a, b, d) and Da Mota Gomes (c).^2^

## References

[1] Da Mota Gomes M, Fontenelle LMC (2007). The emperor Dom Pedro II: his convulsive seizures when a boy. Arq Neuropsiquiatr.

[2] Da Mota Gomes M (2007). The decline of Dom Pedro II's empire and health: neuropathogenic implications. Arq Neuropsiquiatr.

[3] Santos RP, Nardi AE, da Mota Gomes M (2022). Emil du Bois-Reymond and Dom Pedro II: research development on bioelectricity and electric fish. Front Physiol.

[4] Santos RP, Nardi AE, da Mota Gomes M (2023). [How studies on curare contributed to the development of neurophysiological research in Brazil]. Biol Aujourdhui.

[5] Da Mota Gomes M (2021). Louis Pasteur and Dom Pedro II engaged in rabies vaccine development. J Prev Med Hyg.

[6] Santos RP, Nardi AE, da Mota Gomes M (2023). Neurophysiology in between the interests of Dom Pedro II of Brazil, and Charles Brown-Séquard. Rev Bras Neurol.

[7] Nardi AE, Silva AC, Hallak JE, Crippa JA (2013). A humanistic gift from the Brazilian Emperor D. Pedro II (1825-1891) to the Brazilian nation: the first lunatic asylum in Latin America. Arq Neuropsiquiatr.

[8] Aguirre Quiroga S (2024). From Brazil to Brattle Street: the transnational history of emperor Dom Pedro II’s dinner with Henry Wadsworth Longfellow. Am Ninet Century Hist.

[9] Academia Nacional de Medicina (ANM) Cláudio Velho Da Motta Maia (Conde de Motta Maia) [Internet]. http://www.anm.org.br/claudio-velho-da-motta-maia-conde-de-motta-maia/.

[10] Academia Nacional de Medicina (ANM) (2023 Oct 3). Joaquim Candido Soares de Meirelles [Internet]. http://www.anm.org.br/joaquim-candido-soares-de-meirelles-2/.

